# Primary retroperitoneal müllerian adenocarcinoma: a case report

**DOI:** 10.3389/fonc.2024.1364008

**Published:** 2024-06-27

**Authors:** Xiaohui Feng, Ping Zhang, Feng Gao

**Affiliations:** ^1^ Department of Pathology, The Third Hospital of Hebei Medical University, Shijiazhuang, China; ^2^ Department of Radiology, The Third Hospital of Hebei Medical University, Shijiazhuang, China

**Keywords:** müllerian tumor, adenocarcinoma, retroperitoneum, pathology, immunohistochemistry

## Abstract

A 45-year-old woman presented with right hip pain for a month. Imaging results revealed that the left peritoneal mass was accompanied by metastases of the right sciatic branch, lung, and retroperitoneal lymph nodes. A biopsy of the left peritoneal mass was performed. The pathological morphology demonstrated clear cell adenocarcinoma. Immunohistochemical staining revealed a positive expression of keratin7 and PAX8 and a negative expression of keratin20, GCDFP-15, ER, PR, WT1, CDX2, villin, TTF-1, napsin-A, vimentin, calretinin, and GATA3. Finally, the diagnosis of primary retroperitoneal müllerian adenocarcinoma (PRMA) was confirmed. PRMA is a very rare type of primary retroperitoneal tumor. PRMA should be considered for the retroperitoneal mass.

## 1 Introduction

In women, the müller tube forms the fallopian tubes, uterine horn, cervix, and the upper part of the vagina. Although most malignant müllerian adenocarcinomas occur in the uterus, ovaries, fallopian tubes, and vagina, they may occur in rare parts, including the retroperitoneum (1, 2).

Primary retroperitoneal müllerian adenocarcinoma (PRMA) is an extremely rare entity, which often presents in elderly menopausal women and is highly aggressive. Several mechanisms of this clinical entity have been suggested, including retroperitoneal ectopic supernumerary ovarian tissue, retroperitoneal mono-dermal teratoma, coelomic metaplasia, the secondary müllerian system, and retroperitoneal endometriosis (3). The purpose of this study is to report a patient of PRMA and analyze the characteristics of imaging and histomorphology of PRMA to improve the understanding of this tumor.

## 2 Case presentation

This study was conducted under the approval of the Ethics Committee of the Third Hospital of Hebei Medical University, and the protocol was accorded with its standards.

**Figure 1:**
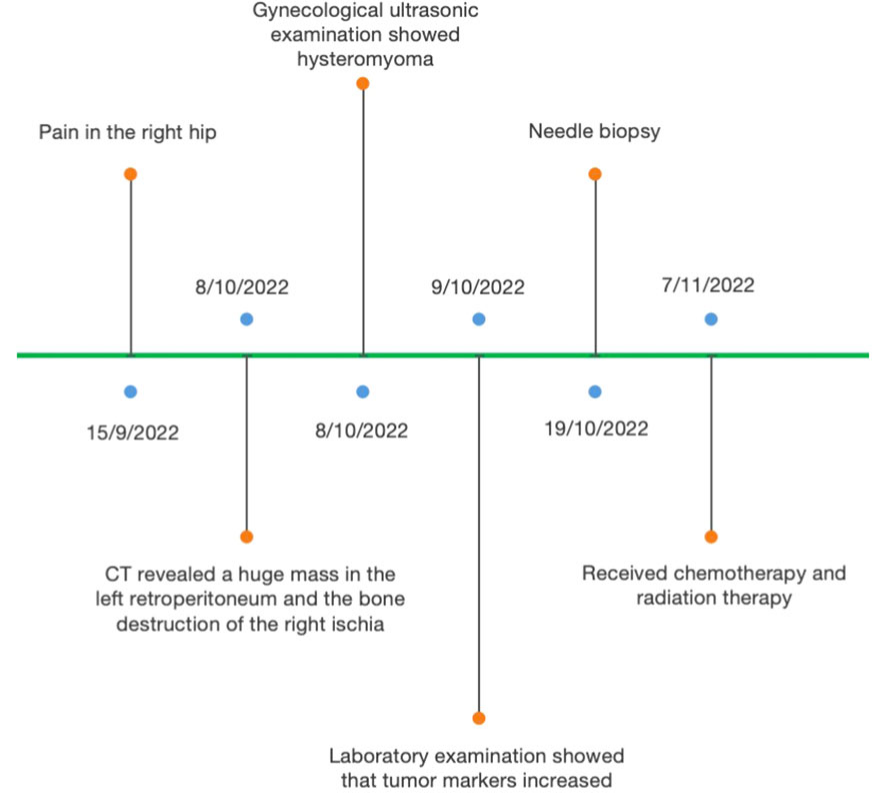
Representative timelines of patient’s relevant data history.

A 45-year-old woman had pain in the right hip for half a month. After admission, the computed tomography (CT) scan incidentally revealed a huge mass in the left retroperitoneum, in addition to the bone destruction of the right ischia. The patient’s menstrual cycle was regular, starting at the age of 14. She was pregnant twice and had given birth twice. No malignant tumors were discovered in this patient before. Results of the gynecological ultrasonic examination showed hysteromyoma, and no abnormality was found in bilateral accessories. The intraepithelial lesions or malignant lesions were not noted by liquid-based thin-layer cell detection. The gastroscopy and enteroscopy results were also normal. The serum CEA increased to 8.39 ng/mL (normal range: <5.2), CA 199 was 1,000 U/mL (normal range: <27), CA125 was 58.29 U/mL (normal range: <35), Cyfra21–1 was 13.4 ng/mL (normal range: <3.3), CA50 was >500 IU/mL (normal range: <25), and CA242 was 184.9 IU/mL (normal range: <20). No abnormality was observed in myeloma monitoring (**Figure 1**).

**Figure 2:**
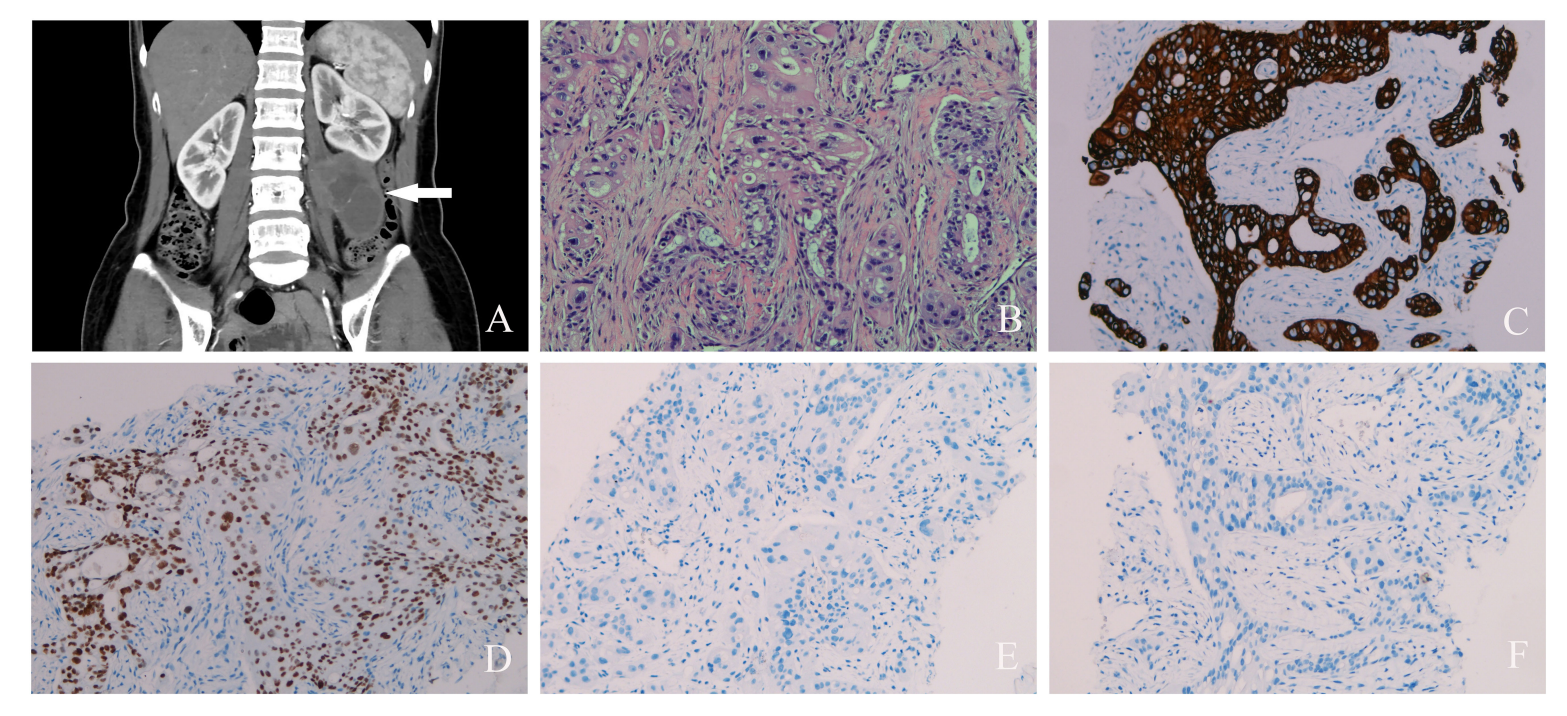
**(A)** Contrast-enhanced computed tomography scan of abdominal pelvic cavity showed a huge cystic and solid mass (white arrow) in the left retroperitoneal space. **(B)** Hematoxylin and eosin staining of primary retroperitoneal müllerian adenocarcinoma (×200). **(C)** Positive keratin7 immunostaining was observed (×200). **(D)** Positive PAX8 immunostaining was observed (×200). **(E)** Negative WT1 immunostaining was observed (×200). **(F)** Negative CDX-2 immunostaining was observed (×200).

The CT scan revealed a 58 × 43-mm retroperitoneal cystic mass with solid components that were indistinguishable from the psoas major and left kidney. Contrast-enhanced CT showed delayed enhancement of solid components and no enhancement of cystic components (**Figure 2A**). Enlarged lymph nodes were scattered in the retroperitoneum. Normality was noted in the liver, gallbladder, pancreas, kidney, pelvis, ureter, and bladder. Multiple small nodules were observed in the lungs, which were highly consistent with pulmonary metastasis (not shown).

A needle biopsy was performed to determine the nature of the retroperitoneal mass. The tumor cells are large and highly atypical, with necrosis in some areas. Most of the cytoplasm was pink, but a small part was transparent. The tissue morphology was glandular and solid and has a cribriform growth pattern. The final pathology result was clear cell adenocarcinoma (**Figure 2B**). The cells were positive for keratin7 and PAX8, and they were negative for keratin20, GCDFP-15, ER, PR, WT1, CDX2, villin, TTF-1, napsin-A, vimentin, calretinin, and GATA3 (**Figures 2C–F**). Finally, PRMA was confirmed.

The patient in this report received six cycles of chemotherapy with carboplatin + paclitaxel, and the tumor bed and the right ischia were irradiated with 45-Gy-intensity modulated volumetric radiotherapy. After the second chemotherapy cycle, the CT scan revealed liver metastasis in this patient. After the fourth chemotherapy cycle, the patient experienced lower back pain and had multiple bone destruction, enlarged pulmonary nodules, and pleural effusion. Adenocarcinoma cells were found in the pleural fluid by pathological examination. Considering the tumor progression of the patient’s condition, targeted treatment with sevotinib was administered. The patient was followed up for 12 months and survived with tumors.

## 3 Discussion

PRMA is an extremely rare entity, which is a diagnosis of exclusion. Primary peritoneal malignant mesothelioma and metastasis of malignant tumors in the urinary system (kidney, bladder) and the female reproductive system (ovaries, fallopian tubes, uterus), digestive system (colon, stomach, pancreas, intestines, gallbladder), and breast and lung must be excluded (4). In this patient, the origin of the breast, digestive system, female reproductive system, and kidney was excluded by medical history, ultrasound, and imaging examination. Pathological histomorphology and immunohistochemistry excluded breast cancer, malignant mesothelioma, and malignant tumors of the digestive system, and female reproductive system.

Patients usually present with specific clinical symptoms, including abdominal distension, pain, and/or palpable masses, but some cases are not specific. The first symptom of our patient was bone pain caused by bone metastasis. The preoperative diagnostic management included ultrasound, CT, and, if necessary, MRI (5). The growth pattern of the tumor was mainly a mixture of solid and cystic components (6). The imaging features were cystic areas with necrosis. In the current case, the characteristics were consistent with the literature.

A variety of different histological subtypes of PRMA, including serous (7), endometrioid (1), clear cell (8), mucinous (4), papillary (2), mixed (serous, mucinous, and endometrioid), and even malignant mixed müllerian tumor (2, 9), has been reported in the literature. The serous, endometrioid, and clear cell histologies were the common types (3). In this report, our PRMA case had poor cell differentiation, and the histopathology was clear cell adenocarcinoma.

The preoperative diagnosis of PRMA is very challenging. Due to the rarity of this tumor, there are no guidelines for the staging, classification, and treatment of PRMA (4). The main treatment methods for PRMA include surgical resection, chemotherapy, and radiation therapy. It is generally recommended that tumor resection should be taken as the main method of treatment in clinical practice, but there is still controversy about the adjuvant treatment of PRMA (2, 3, 7). Patients whose tumors were completely removed had a better prognosis. Due to the strong aggressiveness of this tumor, it is recommended not to break the integrity of the tumor during surgical resection to avoid any peritoneal implantation. Although there is no standard evidence supporting the benefit of adjuvant chemotherapy or radiotherapy because the biological behavior of PRMA resembles that of epithelial-type ovarian carcinoma, platinum-based adjuvant chemotherapy is suggested for these patients. Since metastasis of bone, lung, and lymph nodes has been found in this patient, the tumor was not removed after a puncture biopsy. The patient in this report received palliative chemotherapy with carboplatin + paclitaxel, and external irradiation was selected. After the patient developed the tumor progression, sevotinib was administered.

The differential diagnosis of PRMA should be borne in mind for cases with retroperitoneal mass when the typical adenocarcinoma was noted and the immunohistochemical staining showed positive for keratin7 and PAX8.

## Data availability statement

The raw data supporting the conclusions of this article will be made available by the authors, without undue reservation.

## Ethics statement

The studies involving humans were approved by the Ethics Committee of the Third Hospital of Hebei Medical University. The studies were conducted in accordance with the local legislation and institutional requirements. The participants provided their written informed consent to participate in this study. Written informed consent was obtained from the individual(s) for the publication of any potentially identifiable images or data included in this article.

## Author contributions

XF: Writing – original draft. PZ: Writing – original draft. FG: Writing – review & editing.
